# Curcumin reprograms metabolic pathways and MAPK signaling to exert antidepressant effects

**DOI:** 10.1016/j.bbrep.2025.102399

**Published:** 2025-12-03

**Authors:** Sijin Kong, Lijin Wang, ZiXuan Ren

**Affiliations:** a The First Affiliated Hospital of Bengbu Medical University, Bengbu, 233004, China; b Institute of Mental Health, Bengbu Medical University, Bengbu, 233030, China

**Keywords:** Major depressive disorder, Machine learning, Metabolomics, MAPK signaling, Curcumin

## Abstract

**Background:**

Depression is a prevalent and debilitating mental disorder with limited treatment options. Curcumin, a natural compound with neuroprotective and anti-inflammatory properties, has shown potential antidepressant effects, though the underlying mechanisms remain incompletely understood.

**Methods and results:**

In this study, we investigated the therapeutic effects and molecular mechanisms of curcumin in a chronic unpredictable mild stress (CUMS)-induced rat model of depression. Behavioral assessments, including the sucrose preference test, forced swim test, and open field test, demonstrated that curcumin (50 and 100 mg/kg, orally administered for 21 days) alleviated CUMS-induced anhedonia, behavioral despair, and anxiety-like behaviors, in a dose-dependent manner, with the 100 mg/kg dose exhibiting superior efficacy. Metabolomic profiling of the prefrontal cortex revealed significant metabolic disturbances in CUMS rats, particularly in starch and sucrose metabolism, which were progressively restored by curcumin. Functional enrichment analysis highlighted modulation of neuroinflammation, bioenergetic homeostasis, and signal transduction pathways as key biological processes associated with curcumin's effects. Integrated multi-omics and machine learning approaches identified the MAPK signaling pathway as a central regulatory node. qPCR validation confirmed that curcumin normalized the expression of key MAPK-related genes, including BDNF, EGFR, ERK2, JUN, RAF1, and TNF, with high-dose curcumin consistently showing the most pronounced therapeutic effects.

**Conclusion:**

Our findings demonstrate that curcumin exerts potent antidepressant effects through multi-target mechanisms involving metabolic reprogramming and coordinated regulation of the MAPK signaling pathway. This study provides novel mechanistic insights into curcumin's polypharmacological actions, supporting its potential as a multi-modal therapeutic agent for depression by simultaneously modulating neurotrophic support, inflammatory responses, and intracellular signaling cascades.

## Highlights

• Curcumin reverses CUMS-induced depressive behaviors dose-dependently.
• Metabolomics links curcumin to glutathione/amino acid pathway restoration.
• MAPK signaling (BDNF/EGFR/TNF) is central to curcumin's antidepressant effects.
• Machine learning identifies key targets bridging metabolism and neuroinflammation.
• High-dose curcumin shows superior efficacy in metabolic and transcriptional regulation.

## Graphical abstract

**Figure:**
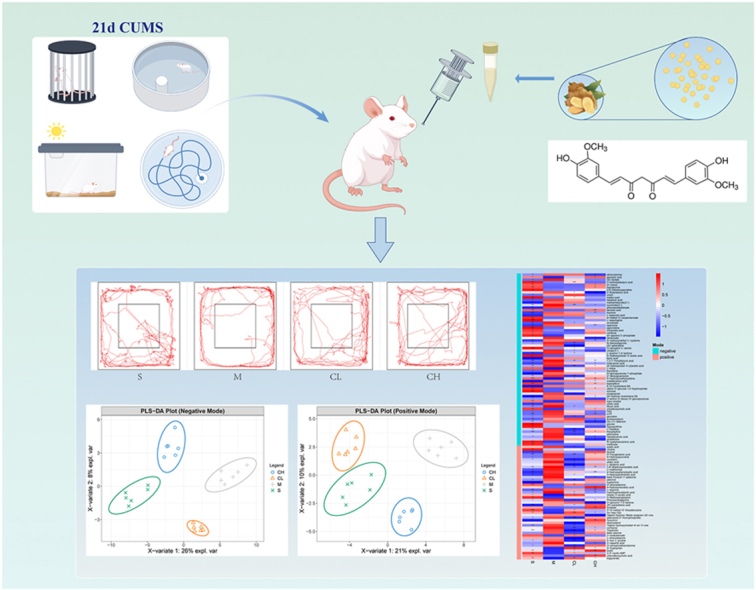

## 1 Introduction

Depression represents a profound and escalating global health crisis, afflicting over 300 million [1] individuals worldwide and constituting a leading cause of disability-adjusted life years (DALYs). Projections indicate it will become the single largest contributor to global disability burden by 2030, surpassing even cardiovascular disease [2]. Despite six decades of intensive pharmaceutical research and substantial economic investment, the therapeutic landscape remains strikingly inadequate. Current first-line pharmacotherapies, predominantly monoaminergic reuptake inhibitors, achieve remission in only 30–40 % of patients after initial treatment, with response rates plateauing over decades. This persistent therapeutic stagnation underscores a critical disconnect: existing drugs largely target singular neurotransmitter pathways, failing to address the intricate, multifactorial pathophysiology now recognized as central to Major Depressive Disorder (MDD) [[3], [4], [5]].

Modern perspectives, informed by systems biology and network medicine, view depression not as a simple shortage of neurotransmitters, but as a complex disorder of interconnected biological systems—resulting from the dynamic and often disrupted interactions among multiple biological networks [6]. Compelling evidence points to the synergistic convergence of neurotrophic deficits (notably reduced Brain-Derived Neurotrophic Factor - BDNF), chronic low-grade neuroinflammation, bioenergetic failure, and mitochondrial dysfunction as core pathophysiological drivers. These elements are not isolated; they form a tightly interconnected web where, for instance, inflammatory cytokines like TNF-α can directly impair mitochondrial function and suppress BDNF signaling, while bioenergetic deficits compromise neuronal resilience and synaptic plasticity [7]. Crucially, conventional antidepressants exhibit limited efficacy in modulating this integrated network, particularly concerning metabolic dysregulation and sustained neuroinflammatory cascades, highlighting an urgent need for novel therapeutic strategies capable of simultaneous, multi-target engagement – a paradigm known as polypharmacology [8].

Natural products, with their inherent chemical complexity and evolutionary history of interacting with multiple biological targets, offer a promising avenue for developing such polypharmacological agents. Among these, curcumin (diferuloylmethane), the principal bioactive curcuminoid derived from *Curcuma longa* (turmeric), has garnered significant scientific interest [9]. Its well-documented pharmacological profile encompasses potent anti-inflammatory, antioxidant, and neuroprotective properties – activities directly relevant to the core pathophysiological axes of depression [10]. Preclinical studies suggest curcumin may exert antidepressant effects through diverse mechanisms, including modulation of monoaminergic neurotransmission (serotonin, dopamine), inhibition of monoamine oxidase (MAO), enhancement of BDNF expression and signaling [11], and suppression of pro-inflammatory cytokines (IL-1β, IL-6, TNF-α). However, a critical limitation persists: the vast majority of investigations have focused on isolated molecular pathways or endpoints. This reductionist approach fails to capture the holistic, systems-level impact of curcumin within the complex, interconnected biological network underlying depression. The precise systemic mechanisms and the comprehensive molecular landscape through which curcumin exerts its potential therapeutic effects remain incompletely resolved, representing a significant knowledge gap hindering its rational development and clinical translation.

The advent of sophisticated multi-omics technologies (transcriptomics, proteomics, metabolomics) provides an unprecedented opportunity to dissect the complex, system-wide actions of natural compounds like curcumin within relevant disease models. These approaches move beyond single-target analyses, enabling the mapping of comprehensive molecular networks and identifying key regulatory nodes. Chronic Unpredictable Mild Stress rodent model, which through prolonged (typically 3–8 weeks) exposure to varied, unpredictable stressors, reliably recapitulates chronic core behavioral and physiological features of human depression, including anhedonia, despair, and metabolic alterations, serves as a robust platform for such investigations [12]. While preliminary evidence suggests curcumin's efficacy in CUMS models, a systematic, multi-omics interrogation of its effects on the intertwined metabolic, inflammatory, and neurotrophic pathways – particularly within the context of the critical MAPK signaling nexus – is lacking.

Therefore, to address these critical gaps, the present study employs an integrated multi-omics strategy to comprehensively elucidate the polypharmacological mechanisms of curcumin in the CUMS-induced depression model. We hypothesized that curcumin's therapeutic efficacy stems from its ability to concurrently modulate interconnected pathophysiological networks, with a specific focus on metabolic reprogramming and MAPK pathway regulation. The Mitogen-Activated Protein Kinase (MAPK) signaling pathway, a highly conserved cascade involving sequential phosphorylation of kinases such as RAF, MEK, and ERK, plays a pivotal role in regulating neuronal plasticity, survival, and inflammatory responses [13]. Dysregulation of this pathway, particularly involving key nodes like BDNF, RAF1, and downstream effectors such as JUN, has been increasingly implicated in the pathophysiology of Major Depressive Disorder. Our objectives were threefold: (1) To rigorously characterize the dose-dependent behavioral effects of curcumin using standardized tests for anhedonia (sucrose preference test), behavioral despair (forced swim test), and locomotor activity/anxiety (open field test); (2) To define the global metabolic alterations induced by CUMS and identify the specific pathways most significantly modulated by curcumin using untargeted LC-MS metabolomics; (3) To delineate the molecular mechanisms underpinning curcumin's effects, with particular emphasis on its coordinated regulation of the MAPK signaling network – including key components like BDNF, RAF1, EGFR, JUN, and TNF – and associated biological processes such as neuroinflammation, bioenergetic homeostasis, and signal transduction, through integrated pathway and functional enrichment analyses. By systematically mapping curcumin's multi-target actions across behavioral, metabolic, and molecular domains, this study aims to establish a robust mechanistic foundation for its development as a novel polypharmacological intervention targeting the multifaceted pathophysiology of depression.

## 2 Materials and methods

### 2.1 Animals and experimental design

Adult male Sprague-Dawley rats (n = 10/group) were acclimatized for 7 days under standard conditions (12-h light/dark cycle, 22 °C, ad libitum food/water, humidity 45–50 %). Rats were randomized into four groups: (1) Sham (S), (2) Model (M), (3) low-dose curcumin (CL, 50 mg/kg), and (4) high-dose curcumin (CH, 100 mg/kg). Vehicle (0.5 % carboxymethylcellulose) or curcumin suspensions were administered via oral gavage once daily for 21 days, starting 30 min before stress sessions. Except for the Sham group (housed socially, 5/cage), all rats were singly housed to induce social isolation. All animal experiments in this study were conducted in strict accordance with the Guide for the Care and Use of Laboratory Animals published by the National Institutes of Health and in compliance with the ARRIVE guidelines for reporting animal research. The protocols were approved by the Animal Ethics Committee of Bengbu Medical University (Approval No. 2024276).

### 2.2 Chronic unpredictable mild stress

The Chronic Unpredictable Mild Stress (CUMS) model was selected for this study because it effectively recapitulates the multifactorial etiology and core behavioral phenotypes of human depression, including anhedonia and behavioral despair, through prolonged exposure to varied, unpredictable stressors [14]. This model is particularly well-suited for investigating the systemic, multi-target effects of curcumin, as it induces complex and interrelated neurobiological alterations encompassing neuroinflammation, HPA axis dysregulation, oxidative stress, and metabolic disturbances—features that align with the polypharmacological nature of curcumin. In contrast, acute inflammatory models such as LPS-induced depression primarily capture the neuroinflammatory component and may not fully represent the chronic, stress-related pathophysiology that characterizes major depressive disorder in humans. Thus, CUMS provides a more comprehensive and clinically relevant platform for evaluating curcumin's therapeutic potential. A 21-day CUMS protocol combined with social isolation was applied. Eight stressors were randomly assigned daily without repetition >4 times: cage rotation (36 h), water/food deprivation (18/24 h), damp bedding (24 h), cage tilting (45°, 18 h), forced swimming (4 °C, 5 min), heat exposure (45 °C, 5 min), and tail clamping (1 min). Stressors were applied at fixed times to maintain circadian consistency.

### 2.3 Behavioral assessments

Tests were conducted 24 h post-CUMS in sequence: Sucrose Preference Test (SPT) [15], Open Field Test (OFT) [16], and Forced Swimming Test (FST) [17], with ≥2 h intervals. All assessments were performed under dim light/low noise by blinded experimenters.

#### 2.3.1 SPT

After 3-day acclimation (Days 1–3: sucrose/water exposure), rats were deprived of water/food for 24 h (Day 3). On Day 4, 1 % sucrose vs. water intake was measured over 3 h. Preference (%) = (sucrose intake/total fluid intake) × 100.

#### 2.3.2 OFT

Rats explored a 80 × 80 × 40 cm arena (25-square grid) for 5 min. Automated tracking recorded total distance, line crossings, rearing frequency, and central/peripheral movement.

#### 2.3.3 FST

Rats swam in 24–26 °C water for 6 min. Immobility (passive floating) and active swimming were recorded during the final 4 min.

### 2.4 Tissue Collection and metabolomics

Rats were euthanized immediately post-behavioral tests. Cerebral cortices were dissected on ice, snap-frozen in liquid nitrogen, and stored at −80 °C. For untargeted metabolomics [18], 30 mg cortical tissue was homogenized in ice-cold methanol:water (4:1) using zirconia beads (70 Hz, 3 × 20 s). Proteins were precipitated (−20 °C, 1 h), and samples centrifuged (16,000×*g*, 15 min, 4 °C). Supernatants were pooled for QC samples, vacuum-dried, and reconstituted in acetonitrile:water (1:1) before UHPLC-MS analysis.

### 2.5 LC-MS analysis

Metabolites were profiled using an SCIEX Exion LC™-Triple TOF™ 5600+ system with an Atlantis™ Premier BEH C18 AX column (100 × 2.1 mm, 1.7 μm, 40 °C). Mobile phases: A (0.1 % formic acid) and B (acetonitrile). Gradient: 0–1 min (1 % B), 1–10 min (1 %→99 % B), 10–13 min (99 % B), 13–14 min (99 %→1 % B), 14–17 min (1 % B). MS operated in positive/negative ESI modes (GS1/GS2 = 55 psi, CUR = 35 psi, 550 °C, ±5500 V). Full-scan MS (*m*/*z* 100–1250) and IDA-based MS/MS (collision energy ±35 ± 15 eV) were acquired. QC samples were analyzed every six runs.

### 2.6 Data processing

Raw data (.wiff) were converted to mzML via ProteoWizard. XCMS online performed peak detection/alignment. R normalized data (median/Pareto scaling) and performed PCA/PLS-DA (mixOmics). Differentially abundant metabolites were defined by VIP >1 and p < 0.05 (*t*-test). Metabolites were annotated via HMDB/METLIN (mass error <10 ppm) or elemental composition.

### 2.7 Identification of core regulatory genes and functional pathways

To identify potential molecular targets linking curcumin's metabolic effects to depression pathophysiology, we integrated metabolomics-predicted target genes with the GEO database (GSE98793) [19] to assess their expression in depression-relevant brain tissues. Core regulators were identified through a consensus machine learning approach: least absolute shrinkage and selection operator (LASSO) regression [20] and extreme gradient boosting (XGBoost) [21]were independently applied for feature selection using the glmnet and xgboost packages in R. Genes selected by both algorithms were defined as core regulatory nodes.

Gene Ontology (GO) [22] and Kyoto Encyclopedia of Genes and Genomes (KEGG) [23] pathway enrichment analyses were performed on the core gene set using the clusterProfiler R package. Enriched biological processes, molecular functions, cellular components, and signaling pathways were visualized using dot plots and pathway maps.

### 2.8 Quantitative real-time PCR validation

Total RNA was extracted using TRIzol reagent, and cDNA synthesized from 5 μg RNA. qPCR was performed using SYBR Green on an ABI 7500 system with thermal cycling: 95 °C for 5min; 40 cycles of 95 °C for 10s and 60 °C for 30s.Primers (Supplementary Table 1) were designed via PrimerBank. Expression calculated via 2^−ΔΔCt^ (GAPDH reference).

## 3 Results

### 3.1 Curcumin alleviates CUMS-induced depression-like behaviors

To investigate the therapeutic potential of curcumin in mitigating depression-like behaviors induced by CUMS, we conducted a comprehensive behavioral analysis incorporating sucrose preference testing, forced swimming testing (FST), and open field testing.

**Fig. 1:**
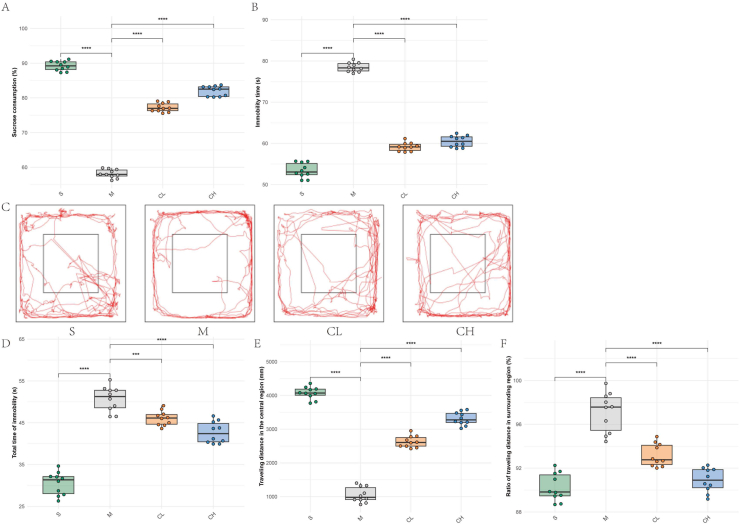
Curcumin ameliorates CUMS-induced depression-like behaviors in rodent models (A) Sucrose preference test (SPT). (B) Immobility time in forced swim test (FST). (C) Representative trajectories from the open field test (OFT). (D) Quantification of total immobility time during OFT. (E) Traveling distance in the central region of the open field. (F) Ratio of traveling distance in surrounding regions. Data are presented as mean ± SEM (n = 10 per group). ∗p < 0.05, ∗∗p < 0.01, ∗∗∗p < 0.001, ∗∗∗∗p < 0.0001 vs. Model; one-way ANOVA followed by Tukey's post hoc test.

SPT data (Fig. 1A) revealed a marked reduction in sucrose consumption in Model rats relative to Sham controls. Curcumin administration elicited dose-dependent recovery, with CL and CH groups exhibiting significant improvements. Notably, the CH group demonstrated superior restoration compared to CL.

FST outcomes (Fig. 1B) highlighted prolonged immobility in Model rats versus Sham. Both curcumin doses reduced immobility duration, with CH showing enhanced efficacy over CL.

OFT trajectory analysis (Fig. 1C) visually distinguished Sham rats' exploratory central zone behavior from Model rats’ peripheral confinement. Quantitative OFT metrics (Fig. 1D–F) further elucidated: (1) Total immobility time was significantly higher in Model versus Sham. Curcumin treatment attenuated this parameter in CL and CH groups, with CH exhibiting greater reduction than CL; (2) Central zone traveling distance, reflective of anxiety-like behavior, was drastically diminished in Model compared to Sham. Curcumin reversed this deficit in CL and CH groups, with CH demonstrating superior recovery; (3) The proportion of traveling distance in surrounding regions (Fig. 1F) was elevated in Model versus Sham. Curcumin treatment normalized this metric in CL and CH groups, with CH differing significantly from CL.

Collectively, these results demonstrate that curcumin mitigates CUMS-induced depressive phenotypes through dose-dependent mechanisms, evidenced by restored anhedonia, reduced behavioral despair, and alleviated anxiety-like responses. The higher efficacy of 100 mg/kg curcumin underscores its potential as a promising intervention for depression management.

### 3.2 LC-MS-based metabolomics uncovers the metabolic basis of Curcumin's therapeutic effects in depression

**Fig. 2:**
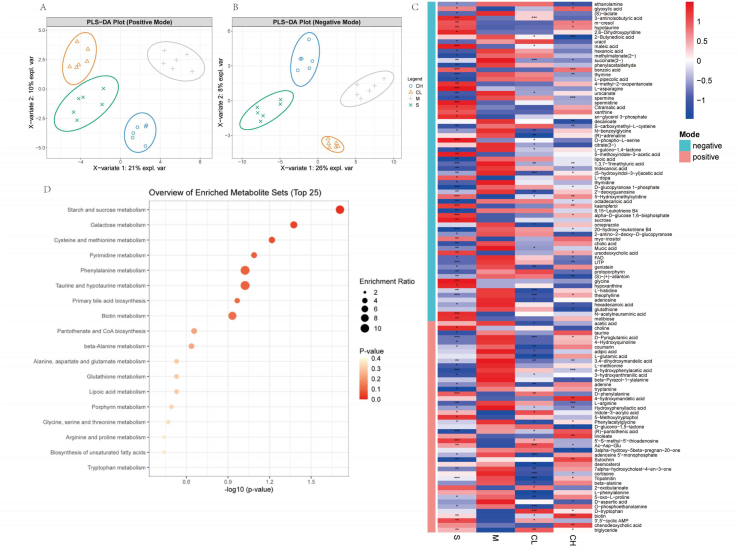
LC-MS-based metabolomics reveals curcumin's modulation of CUMS-induced metabolic perturbations in brain tissue. PLS-DA score plot (positive ion mode, A; negative ion mode, B) showing metabolic profile separation between groups. (C) Heatmap of significantly dysregulated metabolites across experimental groups. (D) Bubble plot of enriched metabolic pathways based on differential metabolite analysis.

Metabolomic characterization (Fig. 2A–B) revealed profound metabolic dysregulation induced by CUMS. PLS-DA models in positive and negative ionization modes demonstrated robust separation between Sham and Model groups, validating the success of CUMS in eliciting metabolic perturbations. Notably, curcumin-treated groups exhibited partial restoration toward Sham-like metabolic profiles, with CH demonstrating greater resilience in reversing CUMS-induced alterations. Heatmap (Fig. 2C) showed 83 significantly dysregulated metabolites (VIP>1, p < 0.05). Our analysis (Fig. 2D) revealed that the glutathione metabolism pathway demonstrated the most robust enrichment, with significant perturbations observed in six critical metabolites including glutathione, glycine, l-glutamic acid, 5-oxo-l-proline, and spermidine. The glycine, serine, and threonine metabolism pathway also exhibited significant enrichment, characterized by alterations in choline, glycine, O-phospho-l-serine, 2-oxobutanoate, and glyoxylic acid. Similarly, the arginine and proline metabolism pathway showed substantial enrichment, with significant changes detected in l-arginine, spermidine, spermine, l-glutamic acid, and glyoxylic acid. Additional pathways including taurine and hypotaurine metabolism, cysteine and methionine metabolism, and glycerophospholipid metabolism also displayed notable enrichment trends. At the individual metabolite level, ethanolamine levels were significantly lower in the Sham group compared to the Model group, and high-dose curcumin intervention effectively normalized this dysregulation. Similarly, glyoxylic acid levels were significantly elevated in the Sham group relative to the Model group, and high-dose curcumin treatment further restored this metabolite toward Sham levels. Collectively, these findings indicate that curcumin, particularly at higher doses, exerts antidepressant effects through coordinated regulation of multiple interconnected metabolic pathways, with glutathione metabolism, amino acid metabolism, and phospholipid metabolism representing key therapeutic targets. This metabolic reprogramming likely contributes to the restoration of CUMS-induced metabolic disturbances, providing compelling mechanistic evidence for curcumin's potential as a multi-target therapeutic agent for depression management.

### 3.3 Machine learning-based identification of core regulators mediating Curcumin's antidepressant effects

**Fig. 3:**
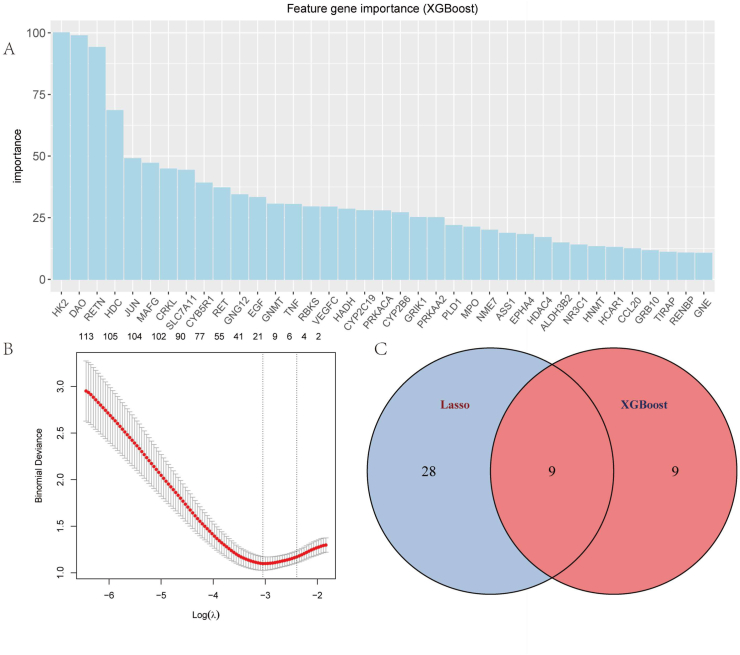
Machine learning-driven feature selection identifies key genes associated with curcumin's antidepressant effects. (A)XGBoost feature importance ranking. (B) LASSO regression analysis. (C) Venn diagram comparing gene sets.

To identify core regulators involved in the therapeutic effects of curcumin on depression-like behaviors, we conducted feature selection analyses using machine learning approaches. Gene intersection analysis revealed 886 genes overlapping between metabolomics-predicted genes (935) and the depression-related transcriptomic dataset (GSE98793). We applied LASSO and XGBoost algorithms independently for feature selection from these 886 genes (see Fig. 3A, B).

LASSO regression analysis (Fig. 3B) demonstrated the relationship between deviance importance and regularization parameter (Log(λ)), showing decreasing deviance importance as Log(λ) increased from −6 to −2. Feature importance ranking from XGBoost (Fig. 3A) identified HK2 as the most significant gene (importance score near 100), followed by DAO, RET, HDC, JUN, and other genes in decreasing order of importance, with GNE showing the lowest importance (near 0).

Venn diagram analysis (Fig. 3C) revealed that LASSO selected 28 genes while XGBoost selected 18 genes, with 9 genes common to both methods. LASSO exclusively identified 19 genes and XGBoost uniquely identified 9 genes. The consensus genes from both algorithms, totaling 46 genes, were selected as potential core regulators for further analysis. This machine learning framework enabled us to identify key targets that might mediate the antidepressant effects of curcumin.

### 3.4 Functional enrichment analyses reveal Curcumin's multi-Scale mechanisms in depression pathogenesis

To elucidate the functional roles of curcumin-regulated core genes in depression pathophysiology, we conducted comprehensive GO and KEGG pathway enrichment analyses on 46 machine-learning prioritized genes.

**Fig. 4:**
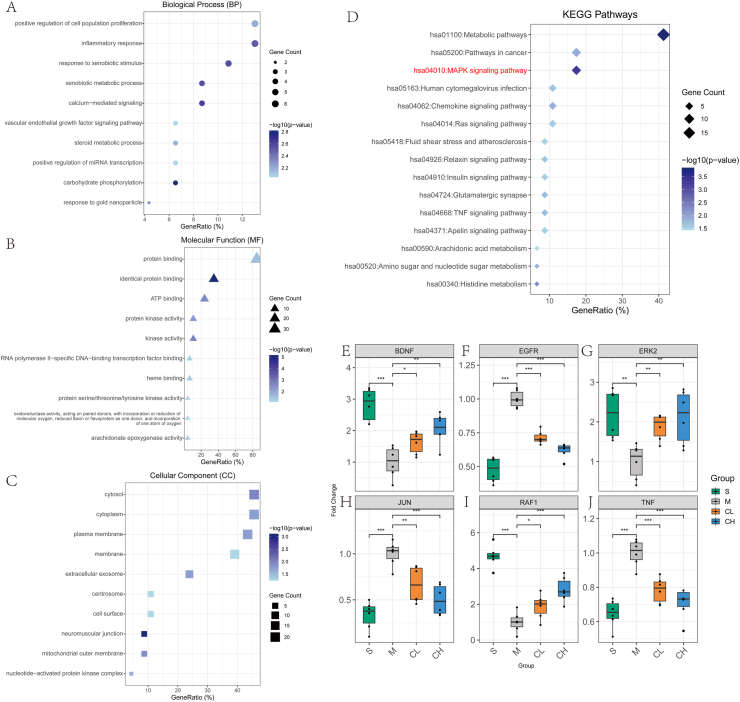
Functional enrichment and MAPK pathway analysis reveal curcumin's multi-target mechanisms in depression. (A) GO Biological Process. (B) GO Molecular Function. (C) GO Cellular Component. (D) KEGG Pathways. qPCR analysis of BDNF (E), EGFR (F), ERK2 (G), JUN (H), RAF1 (I), TNF (J). Data are presented as mean ± SEM (n = 10 per group). ∗p < 0.05, ∗∗p < 0.01, ∗∗∗p < 0.001, ∗∗∗∗p < 0.0001 vs. Model; one-way ANOVA with Tukey's post hoc test.

Biological Process (BP) enrichment (Fig. 4A) revealed robust associations with "positive regulation of cell population proliferation", "inflammatory response", and "xenobiotic metabolic process". Notably, terms related to neurovascular crosstalk, such as "vascular endothelial growth factor signaling pathway", and steroid hormone regulation ("steroid metabolic process") emerged as key processes, highlighting curcumin's dual action on immune modulation and neuroendocrine homeostasis.

Molecular Function (MF) profiling (Fig. 4B) emphasized "protein binding" as the dominant functional attribute, involving hub genes like histone deacetylase 4 (HDAC4) and protein kinase C alpha (PRKACA). Enrichment of "protein kinase activity" and "ATP binding" further implicated enzymatic catalysis and signal transduction cascades in curcumin's mechanism.

Cellular Compartment (CC) distribution (Fig. 4C) demonstrated preferential localization of core genes within "cytosol" and "plasma membrane", with notable representation in "extracellular exosome" and "mitochondrial outer membrane". This spatial organization suggests mitochondrial bioenergetics disruption and exosome-mediated intercellular communication as critical nodes in curcumin's therapeutic axis.

KEGG pathway mapping (Fig. 4D) identified "metabolic pathways" as the most significantly enriched category, housing 19 core genes including histidine decarboxylase (HDC) and aldo-keto reductase family 1 member C3 (AKR1C3). Concurrent enrichment of "MAPK signaling pathway" and "TNF signaling pathway" underscored curcumin's ability to integrate metabolic rewiring with canonical pro-inflammatory cascades.

Collectively, these data reveal a multi-faceted molecular framework wherein curcumin exerts antidepressant effects through coordinated modulation of neuroinflammation, bioenergetic homeostasis, and signal transduction networks, providing a mechanistic rationale for its potential as a polypharmacological intervention in depression.

### 3.5 Curcumin restores MAPK pathway homeostasis via coordinated BDNF, EGFR, and TNF modulation

Our results revealed significant alterations in the expression profiles of these key pathway components following CUMS exposure and subsequent curcumin intervention. Specifically, we observed that CUMS-induced depression models exhibited profound molecular dysregulation characterized by significant BDNF suppression (Fig. 4E), pathological EGFR overexpression (Fig. 4F), ERK2 downregulation (Fig. 4G), pro-inflammatory JUN elevation (Fig. 4H), RAF1 repression (Fig. 4I), and chronic stress-driven TNF upregulation (Fig. 4J). Crucially, the regulatory effects of curcumin on all measured MAPK pathway components exhibited a clear dose-dependent relationship. Specifically, the restoration of BDNF and RAF1 expression, as well as the suppression of EGFR, JUN, and TNF, were significantly more pronounced in the high-dose group (CH, 100 mg/kg) compared to the low-dose group (CL, 50 mg/kg) (Fig. 4E–J). This dose-proportional response was consistent across both behavioral outcomes and molecular parameters, suggesting that higher curcumin doses confer enhanced therapeutic efficacy in the CUMS-induced depression model. BDNF expression, which is critical for neuronal survival and synaptic plasticity, was partially restored by low-dose curcumin and more robustly normalized by high-dose treatment, with the high-dose group approaching Sham baseline levels. Similarly, pathological EGFR overexpression was significantly mitigated by both curcumin doses, with high-dose treatment demonstrating superior suppression compared to low-dose, as confirmed by significant inter-group differences. ERK2 downregulation in CUMS models was rescued by low-dose curcumin and further enhanced by high-dose treatment, with high-dose curcumin bringing ERK2 expression significantly closer to Sham values. The pro-inflammatory transcription factor JUN, which was markedly elevated in depression models, was dose-dependently attenuated by curcumin treatment, with high-dose administration exhibiting significantly greater efficacy than low-dose. RAF1 expression, which was severely repressed in depression models, showed substantial recovery with low-dose curcumin and was nearly normalized with high-dose treatment, approaching Sham group levels. Additionally, TNF upregulation driven by chronic stress was significantly reduced by both curcumin doses, with high-dose treatment achieving greater suppression than low-dose. Collectively, these transcriptional findings demonstrate that curcumin exerts comprehensive regulatory effects on the MAPK signaling pathway through coordinated modulation of neurotrophic factors (BDNF and RAF1), receptor tyrosine kinases (EGFR), and inflammatory mediators (JUN and TNF), with high-dose curcumin consistently demonstrating superior therapeutic efficacy across all measured parameters. The dose-dependent restoration of BDNF and RAF1 expression coupled with the suppression of EGFR, JUN, and TNF provides compelling molecular evidence for curcumin's antidepressant mechanisms, revealing its capacity to rebalance multiple interconnected signaling nodes within the MAPK pathway. This comprehensive transcriptional evidence aligns with and extends our prior multi-omics findings, reinforcing curcumin's potential as a polypharmacological intervention for depression that simultaneously targets neurotrophic support, signal transduction, and inflammatory pathways, thereby addressing the multifactorial nature of depression pathophysiology through a systems-level approach.

## 4 Discussion

Our comprehensive investigation demonstrates that curcumin exerts potent antidepressant effects against CUMS-induced depression through a multi-system regulatory mechanism that simultaneously targets metabolic reprogramming, neuroinflammatory pathways, and MAPK signaling cascades [24]. Our study provides novel and comprehensive evidence that curcumin's dose-dependent therapeutic efficacy is mediated through the coordinated and simultaneous modulation of glutathione metabolism, amino acid homeostasis, and key components of the MAPK signaling pathway. This integrated, systems-level framework significantly advances our understanding of how natural products can target the multifactorial nature of depression. Unlike conventional monoaminergic antidepressants that target single neurotransmitter systems, curcumin's polypharmacological profile addresses the multifactorial nature of depression through simultaneous regulation of interconnected biological networks, offering a paradigm shift in developing multi-target therapeutic strategies for complex neuropsychiatric disorders.

The metabolomics data reveal that curcumin, particularly at 100 mg/kg, effectively normalizes CUMS-induced metabolic dysregulation across multiple interconnected pathways. The glutathione synthesis pathway demonstrates coordinated yet imbalanced changes across experimental groups. l-glutamic acid, a critical substrate for glutathione synthesis, shows significantly elevated levels in the CL group compared to S, indicating potential upregulation of the initial steps of glutathione biosynthesis. Concurrently, glycine - another essential substrate - demonstrates a significant decrease in the M group compared to S, creating a substrate imbalance that may impair efficient glutathione production despite elevated glutamate availability.

This substrate imbalance is further complicated by the significant accumulation of 5-oxo-l-proline in both M and CL groups. 5-oxo-l-proline represents an intermediate in the gamma-glutamyl cycle, and its accumulation typically indicates disruption in the glutathione recycling pathway. Glutathione (GSH) itself shows a complex pattern with a significant decrease in the CH group compared to S, suggesting depletion of the reduced, active form of this critical antioxidant.

The relationship between glutathione metabolism and depression is well-documented, with research indicating that reduced levels of GSH specifically in the occipital region of patients with MDD provide support for the role of oxidative stress in depression pathophysiology [25]. This finding is particularly relevant as oxidative stress and alterations in GSH metabolism of the brain have been connected with many neurological conditions.

Importantly, glutathione appears to have direct antidepressant properties, as demonstrated by studies showing that GSH produces specific antidepressant-like effects in behavioral tests such as the FST. This suggests that interventions targeting glutathione metabolism could represent a novel therapeutic approach. The antidepressant effect of certain compounds, such as Zhi-Zi-Chi Decoction, has been specifically associated with their regulation of glutathione metabolism levels and reduction of oxidative stress damage.

The observed metabolic changes may reflect the body's attempt to counteract oxidative stress, which is increasingly recognized as a key mechanism in depression [26]. Antioxidants act by preventing harmful oxidative effects, which may help to reduce neuroinflammation associated with depressive disorders [27]. This provides a mechanistic link between the observed metabolic alterations and potential antidepressant responses.

Glycine, Serine and Threonine Metabolism shares glycine with the glutathione pathway and includes choline, O-phospho-l-serine, 2-oxobutanoate, and glyoxylic acid. Glycine serves as both a substrate for glutathione synthesis and a neurotransmitter with NMDA receptor modulatory effects. The significant changes in glycine levels across experimental groups suggest potential dual effects on both antioxidant capacity and neurotransmission.

Research indicates that glycine metabolism alterations may influence depression through multiple mechanisms, including modulation of NMDA receptor function and regulation of one-carbon metabolism, which is critical for neurotransmitter synthesis. The connection between platelet COX activity and the severity of depression after antidepressant therapy suggests that these metabolic changes may serve as biomarkers for treatment response.

Arginine and Proline Metabolism intersects with glutathione metabolism through spermidine and l-glutamic acid. Spermidine and spermine (polyamines) have been implicated in neuroplasticity and stress response regulation. The significant changes in these metabolites across groups may reflect adaptations in cellular stress responses that are relevant to depression pathophysiology.

Polyamines like spermidine have been shown to influence neuronal function through multiple mechanisms, including modulation of ion channels and regulation of protein synthesis. The interplay between polyamine metabolism and glutathione pathways suggests a coordinated response to oxidative stress that may be targeted by antidepressant interventions.

Though taurine and Hypotaurine Metabolism contains only two metabolites (hypotaurine and taurine), it represents an important antioxidant system [26]. Taurine has been shown to have neuroprotective effects and modulate GABAergic neurotransmission, both of which are relevant to depression pathophysiology [28].

The significant alterations in taurine metabolism may indicate compensatory mechanisms to counteract oxidative stress in depression. Previous studies have reported both increases and decreases in glutathione-related metabolites in depression, suggesting complex regional and temporal dynamics in the antioxidant response.

Cysteine and Methionine Metabolism provides cysteine, the rate-limiting substrate for glutathione synthesis [29]. The metabolites 5′-S-methyl-5′-thioadenosine, l-methionine, 2-oxobutanoate, and O-phospho-l-serine show significant changes across experimental groups, suggesting alterations in one-carbon metabolism and transsulfuration pathways.

These metabolic shifts may influence depression through multiple mechanisms, including regulation of homocysteine levels (elevated homocysteine is associated with depression), modulation of DNA methylation patterns, and provision of cysteine for glutathione synthesis. The connection between these metabolic changes and antidepressant response warrants further investigation.

Glycerophospholipid Metabolism includes choline, O-phosphoethanolamine, ethanolamine, and sn-glycerol 3-phosphate. Significant alterations in these metabolites suggest changes in membrane phospholipid composition and cholinergic signaling.

Choline metabolism is particularly relevant to depression, as it serves as a precursor for acetylcholine and phosphatidylcholine, both critical for neuronal membrane integrity and neurotransmission [30]. The observed changes may reflect adaptations in membrane fluidity and signaling that could influence antidepressant response.

The comprehensive analysis reveals that the glutathione metabolism pathway does not operate in isolation but intersects with several other significantly altered pathways. This interconnected dysregulation creates a metabolic network effect where disruption in one pathway amplifies disturbances in others, potentially influencing the development and treatment of depression.

The observed metabolic changes across experimental groups may represent both pathological mechanisms and compensatory responses. For instance, the accumulation of 5-oxo-l-proline alongside substrate imbalances suggests impaired glutathione recycling, which would compromise the brain's antioxidant defense system. When glutathione status is altered in specific brain regions, it affects multiple related metabolic pathways and may influence depressive symptomatology.

These findings suggest that therapeutic interventions targeting multiple aspects of glutathione metabolism and related pathways—such as enhancing substrate availability, supporting glutathione recycling, or modulating polyamine metabolism—could potentially augment antidepressant effects. The layered metabolic changes observed provide valuable insights into the biochemical mechanisms underlying depression and highlight glutathione metabolism as a critical pathway for further investigation in depression research.

Our qPCR analyses provide compelling evidence that curcumin's antidepressant effects are mediated through regulation of the MAPK signaling pathway, with high-dose treatment demonstrating superior efficacy across all measured parameters. The profound BDNF suppression in CUMS models represents a hallmark of depression-related synaptic dysfunction, consistent with the neurotrophic hypothesis of depression. Curcumin's dose-dependent restoration of BDNF expression directly links to the observed behavioral improvements, particularly in the sucrose preference test, which measures anhedonia—a core symptom of depression. Equally significant is the pathological EGFR overexpression in depression models, which has not been previously emphasized in depression research. EGFR signaling has recently emerged as a critical regulator of neuroinflammation and glial activation, and its normalization by curcumin provides a novel mechanistic insight into the compound's anti-inflammatory effects. The parallel restoration of ERK2 and RAF1 establishes curcumin's ability to correct the entire MAPK cascade, which is essential for synaptic plasticity and neuronal survival. The dose-dependent attenuation of pro-inflammatory markers JUN and TNF further demonstrates curcumin's dual action on neuronal plasticity and neuroinflammatory processes, addressing two fundamental pathophysiological mechanisms in depression that are often treated as separate entities in conventional therapeutic approaches.

Our findings significantly advance the current understanding of depression mechanisms and treatment in several key ways. Our demonstration of curcumin's effects on glutathione metabolism extends recent findings, who reported redox imbalance in depression but did not establish therapeutic strategies targeting this pathway. The integration of metabolomics with MAPK signaling provides a more complete picture than single-omics approaches that have dominated depression research. Unlike recent studies focusing exclusively on inflammatory markers, our work demonstrates how metabolic, inflammatory, and neurotrophic pathways are interconnected and can be simultaneously targeted—a systems approach that aligns with the emerging network psychiatry framework. This comprehensive perspective addresses a critical limitation in current depression research, which often investigates isolated biological pathways rather than their dynamic interactions.

Despite the robust findings, several limitations warrant consideration. First, our study employed a rodent CUMS model, which, while widely accepted, cannot fully recapitulate the complex etiology of human depression. The translational relevance of our findings requires validation in clinical populations with careful consideration of interspecies differences in metabolism and neurobiology. Second, while the cerebral cortex was selected based on its established role in depression pathophysiology and metabolic dysregulation, this regional focus represents a limitation. Depression involves complex interactions among multiple brain regions with distinct pathophysiological contributions: the hippocampus regulates HPA axis function and exhibits stress-induced atrophy; the amygdala mediates emotional reactivity; the prefrontal cortex governs executive function and emotional regulation; and the nucleus accumbens controls reward processing and anhedonia. Metabolic and molecular alterations in cortical tissue may not fully represent region-specific changes, as different areas may exhibit distinct vulnerability profiles and treatment responses. For example, the hippocampus shows particularly robust BDNF suppression in depression models, while the nucleus accumbens demonstrates dopaminergic dysfunction specifically relevant to anhedonia. Future studies employing region-specific multi-omics profiling across this depression-relevant neural circuitry would provide critical insights into circuit-specific therapeutic mechanisms and potentially identify region-selective biomarkers of treatment response. Third, while our multi-omics approach provides comprehensive insights, the causal relationships between observed molecular changes and behavioral outcomes remain correlational. Future research incorporating targeted genetic or pharmacological interventions would strengthen causal inferences. Finally, the exclusive focus on male subjects, while common in preclinical depression research to avoid hormonal variability, limits the generalizability of findings to female populations, where depression prevalence is higher. Future studies should investigate sex-specific responses to curcumin.

## 5 Conclusion

In summary, this study demonstrates that curcumin exerts potent antidepressant effects in a CUMS-induced rat model of depression through a multi-system mechanism. It concurrently reprograms dysregulated metabolic pathways (notably glutathione and amino acid metabolism) and restores homeostasis of the MAPK signaling pathway by coordinately modulating neurotrophic (BDNF, RAF1), inflammatory (TNF, JUN), and signaling (EGFR, ERK2) components. These effects are dose-dependent, with 100 mg/kg showing superior efficacy. Our findings provide a robust mechanistic foundation for curcumin's development as a novel polypharmacological agent targeting the multifaceted pathophysiology of depression.

## Data availability statement

The original contributions presented in this study are included in the article; further inquiries can be directed to the corresponding author.

## Ethics statement

Ethical approval was granted by the Ethics Committee of The First Affiliated Hospital of Bengbu Medical University.

## Funding information

This study was supported by NatureScot project of Bengbu Medical University (2023byzd061) and 10.13039/501100018628Scientific Research Foundation of Education Department of Anhui Province of China (2023AH051971).

## CRediT authorship contribution statement

**Sijin Kong:** Funding acquisition, Methodology, Writing – original draft, Writing – review & editing. **Lijin Wang:** Methodology, Software, Writing – original draft. **ZiXuan Ren:** Data curation, Methodology, Supervision, Writing – review & editing.

## Declaration of competing interest

The authors have no conflict of interest.

## Data Availability

Data will be made available on request.
