# Supplementary material for: Curcumin reprograms metabolic pathways and MAPK signaling to exert antidepressant effects
Source: Biochem Biophys Rep. 2025 Dec 3;45:102399. doi: 10.1016/j.bbrep.2025.102399 (PMC12719971; doi:10.1016/j.bbrep.2025.102399)
Supplement: Multimedia component 1 [file mmc1.docx]

| **TABLE 1. The sequence of primers of genes** | | |
| --- | --- | --- |
| **Genes** | **Forward** | **Reverse** |
| JUN | GCCACCGAGACCGTAAAGAA | TAGCACTCGCCCAACTTCAG |
| BDNF | AAGTCTGCATTACATTCCTCGA | GTTTTCTGAAAGAGGGACAGTTTAT |
| TNF | AAATGGGCTCCCTCTCATCCAGTTC | TCTGCTTGGTGGTTTGCTACGAC |
| ERK2 | GAGAACATCATCGGCATC | TCAGCAGGAGGTTGGAAG |
| EGFR | TCCCAAAGAAGCCAAGCCGAATG | TGCCTCTTCAATGTCATGCTCCAC |
| RAF1 | CAATGGTTTCGGACTCAA | GCGTCGGTGTTCCAATCT |
| GAPDH | GCCAGTAGACTCCACGACA | GCAAGTTCAACGGACAAG |
